# Supplementary material for: Physics-based parameterisation framework for basal melting in ice-ocean boundary layers over dynamically stable pycnoclines
Source: Commun Earth Environ. 2025 Nov 13;6(1):897. doi: 10.1038/s43247-025-02829-6 (PMC12615261; doi:10.1038/s43247-025-02829-6)
Supplement: Supplementary file 2 — Reporting Summary [file 43247_2025_2829_MOESM2_ESM.pdf]

nature portfolio

Corresponding author(s): *Double-anonymous peer review submissions: write DAPR and your manuscript number here instead of author names.*

Last updated by author(s): *YYYY-MM-DD*

Reporting Summary

Nature Portfolio wishes to improve the reproducibility of the work that we publish. This form provides structure for consistency and transparency in reporting. For further information on Nature Portfolio policies, see our Editorial Policies and the Editorial Policy Checklist.

Statistics

For all statistical analyses, confirm that the following items are present in the figure legend, table legend, main text, or Methods section.

n/a	Confirmed	
☐	☒	The exact sample size (*n*) for each experimental group/condition, given as a discrete number and unit of measurement
☐	☒	A statement on whether measurements were taken from distinct samples or whether the same sample was measured repeatedly
☒	☐	The statistical test(s) used AND whether they are one- or two-sided<br>*Only common tests should be described solely by name; describe more complex techniques in the Methods section.*
☒	☐	A description of all covariates tested
☒	☐	A description of any assumptions or corrections, such as tests of normality and adjustment for multiple comparisons
☒	☐	A full description of the statistical parameters including central tendency (e.g. means) or other basic estimates (e.g. regression coefficient) AND variation (e.g. standard deviation) or associated estimates of uncertainty (e.g. confidence intervals)
☒	☐	For null hypothesis testing, the test statistic (e.g. *F*, *t*, *r*) with confidence intervals, effect sizes, degrees of freedom and *P* value noted<br>*Give P values as exact values whenever suitable.*
☒	☐	For Bayesian analysis, information on the choice of priors and Markov chain Monte Carlo settings
☒	☐	For hierarchical and complex designs, identification of the appropriate level for tests and full reporting of outcomes
☒	☐	Estimates of effect sizes (e.g. Cohen's *d*, Pearson's *r*), indicating how they were calculated

Our web collection on statistics for biologists contains articles on many of the points above.

Software and code

Policy information about availability of computer code

Data collection	The data for this study were generated computationally, not collected from an external source. The datasets were created by running a series of simulations using an Ice-Ocean Boundary Current Model (IOBCM). The simulations were run until they reached a steady state, and the variables were then time-averaged over the final 10 of 90 inertial cycles to create the final datasets.
Data analysis	The data analysis for this study was performed using custom algorithms and software written in MATLAB. This code was used to process the output from the IOBCM simulations, perform calculations, and generate the figures presented in the manuscript. The code used to reproduce the figures and generated datasets is publicly available at https://doi.org/10.5281/zenodo.17051525 . The MATLAB model itself is also available at https://doi.org/10.5281/zenodo.13381662 .

For manuscripts utilizing custom algorithms or software that are central to the research but not yet described in published literature, software must be made available to editors and reviewers. We strongly encourage code deposition in a community repository (e.g. GitHub). See the Nature Portfolio guidelines for submitting code & software for further information.

Data

Policy information about availability of data

All manuscripts must include a data availability statement. This statement should provide the following information, where applicable:

- Accession codes, unique identifiers, or web links for publicly available datasets
- A description of any restrictions on data availability
- For clinical datasets or third party data, please ensure that the statement adheres to our policy

The generated datasets and codes to reproduce the figures in this study are publicly available at https://doi.org/10.5281/zenodo.17051525 and they are free to use

Research involving human participants, their data, or biological material

Policy information about studies with human participants or human data. See also policy information about sex, gender (identity/presentation), and sexual orientation and race, ethnicity and racism.

Reporting on sex and gender	Not applicable as there are no human participants.
Reporting on race, ethnicity, or other socially relevant groupings	Not applicable as there are no human participants.
Population characteristics	Not applicable as there are no human participants.
Recruitment	Not applicable as there are no human participants.
Ethics oversight	Not applicable as the study is a computational modeling and is not subject to human ethics review.

Note that full information on the approval of the study protocol must also be provided in the manuscript.

Field-specific reporting

Please select the one below that is the best fit for your research. If you are not sure, read the appropriate sections before making your selection.

☐ Life sciences ☐ Behavioural & social sciences ☒ Ecological, evolutionary & environmental sciences

For a reference copy of the document with all sections, see nature.com/documents/nr-reporting-summary-flat.pdf

Ecological, evolutionary & environmental sciences study design

All studies must disclose on these points even when the disclosure is negative.

Study description	This is a physics-based modeling study investigating the parameterization of basal melting in z-coordinate ocean models for a stratified water column. The research was motivated by a study (Davis et al., 2023) that found conventional models significantly overestimate basal melting. While the current work focuses on the simple case of a horizontal ice-ocean interface (IOI), it proposes a new, general physics-based parameterization framework for ocean-ice heat transfer. This framework is designed to be versatile and adaptable to more complicated settings in the future, with the presented work laying a useful foundation for subsequent validation and expansion. The study uses an Ice-Ocean Boundary Current Model (IOBCM) to simulate and analyze the processes of heat transfer and mixing within the ice-ocean boundary layer.
Research sample	The study does not use a traditional biological or sociological "sample." Instead, the "sample" is a dataset generated from a range of IOBCM simulations. These simulated data points represent properties of the ice-ocean boundary layer (BL) and include variables such as thermal driving, viscosity, diffusivity, friction velocity, and melting. The simulations vary key model parameters to create a parameter space that includes ambient thermal driving, turbulent transfer coefficient, drag coefficient, far-field current speed, and Coriolis parameter. The model variables are time-averaged over the last 10 of a total of 90 inertial cycles of each simulation to form the datasets.
Sampling strategy	This is not applicable as the study involves computational modeling, not sampling from a population or environment. The "sample" is the entire set of datasets generated from the model simulations, which are created by systematically varying key parameters across a predefined range.
Data collection	Data were "collected" by generating them computationally. The data were produced by running a series of simulations using an Ice-Ocean Boundary Current Model (IOBCM). The simulations were run until they reached a steady state, and the variables were then time-averaged over the final 10 inertial cycles to create the final datasets.
Timing and spatial scale	The timing of the simulations is measured in inertial cycles, with each simulation run for up to 90 inertial cycles until a steady state is reached. Data were time-averaged over the last 10 cycles. The spatial scale is implied by the model's focus on the ice-ocean boundary layer (BL) and the dynamically stable pycnocline. The depth of the BL is a key variable, with the effective thermal

	driving measured at a distance of approximately 0.5 meters from the ice-ocean interface.
Data exclusions	All generated data from the range of IOBCM simulations were included in the analysis.
Reproducibility	The study is designed to be fully reproducible. The model used is based on Jenkins (2021), and the generated datasets and codes used to reproduce the figures are publicly available. The provided DOIs are:<br>• Matlab Code for model: https://doi.org/10.5281/zenodo.13381662<br>• Generated Datasets and Codes for Figures: https://doi.org/10.5281/zenodo.17051525
Randomization	This is not applicable. The study does not involve randomization, as it is a computational modeling study where parameters are systematically varied to explore a defined parameter space.
Blinding	Blinding is not applicable to this study.

Did the study involve field work? ☐ Yes ☒ No

Reporting for specific materials, systems and methods

We require information from authors about some types of materials, experimental systems and methods used in many studies. Here, indicate whether each material, system or method listed is relevant to your study. If you are not sure if a list item applies to your research, read the appropriate section before selecting a response.

Materials & experimental systems

n/a	Involved in the study	
☒	☐	Antibodies
☒	☐	Eukaryotic cell lines
☒	☐	Palaeontology and archaeology
☒	☐	Animals and other organisms
☒	☐	Clinical data
☒	☐	Dual use research of concern
☒	☐	Plants

Methods

n/a	Involved in the study	
☒	☐	ChIP-seq
☒	☐	Flow cytometry
☒	☐	MRI-based neuroimaging

Plants

Seed stocks	We have not used seed stokes in this study.
Novel plant genotypes	NO plant genotypes were produced as a part of this study.
Authentication	We have not used any seed stocks.
